# Intersectional Stigma and Health of People Living with HIV on ART Who Use Alcohol in Southwestern Uganda

**DOI:** 10.1007/s10461-026-05120-7

**Published:** 2026-04-03

**Authors:** Sarah A. Gutin, Esther Atukunda, Fatema Shafie Khorassani, Robin Fatch, Kaku So-Armah, Adah Tumwegamire, Nneka I. Emenyonu, Cristina Espinosa da Silva, Christine Ngabirano, Karsten Lunze, Julian Adong, Winnie Muyindike, Judith A. Hahn

**Affiliations:** 1 Department of Community Health Systems, School of Nursing, University of California, San Francisco, 490 Illinois Street, Floor 9, San Francisco, 94143 USA; 2 Division of Prevention Sciences, Department of Medicine, University of California, San Francisco, San Francisco, USA; 3 Department of Pharmacology/Pharmacy, Mbarara University of Science and Technology, Mbarara, Uganda; 4 Department of Biostatistics, Boston University School of Public Health, Boston, MA USA; 5 Department of Medicine, University of California, San Francisco, San Francisco, CA USA; 6 Department of Medicine, Boston Medical Center and Boston University Chobanian and Avedisian School of Medicine, Boston, MA USA; 7 Global Health Collaborative, Mbarara University of Science and Technology, Mbarara, Uganda; 8 Department of Internal Medicine, Mbarara Regional Referral Hospital, Mbarara, Uganda; 9 Department of Epidemiology, University of California, San Francisco, San Francisco, CA USA

**Keywords:** Intersectional stigma, TB stigma, Alcohol stigma, HIV stigma, Uganda

## Abstract

HIV, TB, and alcohol use are stigmatized conditions that lead to poor care engagement and health outcomes. Stigmatized traits can operate independently or be intersectional. We examined the relationships between intersectional HIV-, TB-, and alcohol-related stigma on poorer perceived health among people with HIV (PWH) receiving antiretroviral therapy in a study examining TB infection risk among PWH in HIV care in southwestern Uganda (2022–2023). We used proportional odds models to examine associations between high intersectional HIV, TB, and alcohol stigma (defined as above median scores on validated scales) and the outcome of poorer perceived health. Among 379 PWH, 12% described their health status as fair/poor. High intersectional HIV and alcohol stigma was associated with increased odds of poorer perceived health (adjusted odds ratio [aOR] = 1.62; 95% CI: 1.04–2.52), but we found no associations between other HIV, TB, and alcohol stigma intersections and this outcome. We found a significant interaction between intersectional HIV and alcohol stigma and marital status (Wald χ^2^ = 5.02, *p* = 0.03), and upon stratification, high intersectional HIV and alcohol stigma was associated with an increased odds of poorer perceived health among unmarried participants (aOR = 2.54; 95% CI: 1.33–4.86; *p* < 0.01) but not among married participants (aOR = 1.05; 95% CI: 0.56–1.95; *p* = 0.88). High intersectional HIV and alcohol stigma was associated with poorer perceived health among PWH in care, particularly among unmarried persons. Given the possible benefits of partner support, interventions that strengthen social support for unmarried persons may help mitigate the negative health impact of intersectional stigma.

## Introduction

HIV, TB, and alcohol use are all socially stigmatized conditions that can lead to poor care engagement and health outcomes [1–4]. Stigma was defined by Erving Goffman in 1963 as “a deeply discrediting attribute” [5] that is socially constructed and relational.

Although common, alcohol use disorders are highly stigmatized both socially and within healthcare settings [6–8]. As a result of this and other barriers, only a small number of people who need help seek services [9]. In particular, negative attitudes and stigma from healthcare providers in both Western countries and sub-Saharan Africa contribute to poor uptake and engagement with healthcare services by those with substance use disorders [7, 8, 10, 11]. In Uganda where the HIV prevalence among those aged 15–49 years is 5.1% [12], 22% of men and 8% of women living with HIV engage in high or very high alcohol consumption [13].

HIV-related stigma and discrimination adversely affect the health and quality of life of PWH and lead to a host of poor health-seeking behaviors and poor physical and mental health outcomes [14]. HIV stigma has been associated with hazardous drinking among PWH [15–18] and both substance use and related stigma are barriers to seeking care and treatment, and adhering to ART medications [11]. Studies have also suggested that PWH may use alcohol and other drugs to cope with HIV stigma [10].

TB stigma and HIV stigma often intersect. Studies in South Africa, Namibia, and Lesotho have found that TB and HIV stigma act individually and together [1, 19–21] to create isolation and exclusion and can impair health-seeking behaviors and lead to care disengagement [4, 19]. Furthermore, a study in Lesotho found that unhealthy alcohol use was significantly associated with greater TB and HIV stigma [21]. Uganda has a high HIV/tuberculosis (TB) co-infection burden, with an estimated TB incidence of 200 persons per 100,000 in 2019 [22]. TB disease is more common among those living with HIV and exhibiting unhealthy alcohol use, and is the leading cause of death among PWH [22].

As noted above, stigma can be intersectional, operate at many levels, and be related to multiple, synergistic and overlapping identities, traits, or illnesses [23, 24]. Intersectionality is a critical framework that examines multiple types of marginalization that can affect a person at the same time – or at different times- and is often multiplicative [25, 26]. Therefore, intersectionality allows for examining multiple factors or stigmatized identities that can affect one’s place in the social order, their power, and agency [25]. Intersectionality also allows for the examination of how multiple intersecting identities can affect privilege and how systems of oppression are perpetuated at the broader social and structural level [24, 27, 28]. While studies have examined the intersectional nature of HIV and alcohol stigma [8, 10, 29], TB and HIV stigma [19–21], and TB, HIV, and substance use [30], to our knowledge none have specifically assessed the overlapping and intersectional nature of HIV, TB, and alcohol stigma together.

In the context of intersectional stigma, supportive relationships may moderate the relationship between HIV stigma and/or TB stigma and harmful alcohol use. Various studies have found that partner support is critical to enacting health enhancing behaviors such as HIV testing, HIV status disclosure, and ART adherence [31–35]. Few studies have examined the impacts of partner support on TB treatment adherence [36] or alcohol reduction [37–39]. While stigma can be the result of or create fears of discrimination and abandonment and stress in relationships [40–42], healthy couple relationships may help protect against the harms of stigma and negative health outcomes. Therefore, marital status, as a proxy for partner support, may have a positive impact on experiences of stigma. By examining whether marital status has a moderating effect on the relationship between stigma and perceived health, we can determine if intervening at the couple-level might be an effective way to reduce the effect of stigma on PWH.

While they are often analyzed in isolation, different forms of stigma usually do not manifest in isolation, and the effects of more than one type of marginalization or stigma can be more than just the additive effect of the separate stigmas. An increasing number of studies have examined how different forms of stigma together can intersect and impact health outcomes [10, 29, 43]. Recent studies from South Africa and Russia have found that HIV and alcohol or substance use stigmas were linked with increased depressive symptoms, providing findings to suggest that multiple layers of stigma together, i.e., intersectional stigma, can worsen mental health outcomes for PWH [29, 43]. While some literature has considered the intersectional nature of HIV stigma and alcohol stigma [44], there is little research looking at the intersectional and possible compounding effects of HIV stigma, TB stigma, and alcohol stigma. Hence, among a sample of people with HIV on ART who reported alcohol use in southwestern Uganda, we sought to: (1) examine the level of HIV, TB, and alcohol stigma scores overall and by gender, (2) examine the intersectional relationships between HIV, TB, and alcohol stigma on poorer perceived health, and (3) assess whether these relationships are moderated by marital status.

## Methods

### Study Setting and Participants

We conducted this analysis using baseline data from the TB Risk by Alcohol Consumption (TRAC) Study, a prospective cohort study that examined the risk of acquiring TB infection and of incident active TB disease among PWH with heavy alcohol use. The study was conducted at the Immune Suppression Syndrome (ISS) HIV clinics at both the Mbarara University of Science and Technology (MUST) and Mbarara City Clinic (MCC) in Southwestern Uganda. This study is part of the Uganda-Russia-Boston Alcohol Network for Alcohol Research Collaboration on HIV/AIDS (URBAN ARCH) Consortium.

Patients were eligible for the TRAC study if they were adults (≥ 18 years old) living with HIV who had tested tuberculin skin test (TST) negative in the screening activities for one of two prior studies – the Alcohol Drinkers’ Exposure to Preventive Therapy for Tuberculosis (ADEPTT) [45] and the Drinkers’ Intervention to Prevent Tuberculosis (DIPT) studies [46, 47], resided in the study catchment area (< 60 km or < 2 h travel), and had the ability to give informed consent. In addition, participants for the TRAC study were purposively enrolled so that about 25% of PWH had no alcohol use, 25% had low to moderate alcohol use, and 50% had high-risk alcohol use. For this analysis, we examined only those with available data on the three stigma scales of interest (HIV, TB, and alcohol stigmas).

### Study Procedures

Potential participants were led through the informed consent process, administered a survey, screened for latent TB infection using TST, and assessed for symptoms of active TB. The informed consent process involved re-consenting participants who took part in the screening activities for the ADEPTT or DIPT studies. Participants were told during the consent process that they were being asked to take part in the study because they might be at risk for TB infection.

Face-to-face surveys were administered by Ugandan research assistants from April 2022 to October 2023. The participants were interviewed in English or Runyankole, depending on the participant’s preference. The survey included demographic variables, self-reported alcohol consumption, measured by the 3-question Alcohol Use Disorders Identification Test-Consumption (AUDIT-C [48], modified to cover the prior 3 months), self-reported perceived health status, and HIV, TB, and alcohol-related stigmas.

Venous blood draws were conducted, and samples were collected and pipetted onto filter paper to prepare dried blood spots (DBS) to measure phosphatidylethanol (PEth), a quantitative biomarker of recent alcohol consumption with high sensitivity and specificity for detecting any and high-risk alcohol use over the past 2 to 4 weeks [49]. PEth ≥ 50 ng/ml is consistent with unhealthy drinking [50] and PEth ≥ 200 ng/ml is consistent with high-risk drinking [51]. The DBS cards were tested for PEth (16:0/18:1 homologue, with an 8 ng/ml lower limit of quantification) at the United States Drug Testing Laboratories (USDTL, Des Plaines, IL) [48].

### Measures

Perceived health status: Perceived health status was assessed using one question from the Medical Outcomes Study HIV Health Survey (MOS-HIV) [52, 53] that was adapted for Uganda [54]. The question asked, “In general, would you say your health is excellent, very good, good, fair, or poor?” This variable was treated as an ordinal variable with 4 categories (excellent, very good, good, poor/fair) such that higher scores indicate poorer perceived health status.

HIV stigma scale: HIV stigma was assessed using a 10-item scale [55] covering four dimensions of HIV stigma: personalized stigma (3 items), disclosure concerns (2 items), negative self-image (3 items), and perceived public attitudes about people living with HIV (2 items). Scale items were summed such that higher values denoted higher levels of HIV stigma. The Cronbach’s alpha value for the HIV stigma scale was 0.77, indicating moderate internal consistency.

TB stigma scale: TB stigma was assessed using a 23-item scale that measures stigma associated with TB [56]. The scale captures two constructs: community perspectives towards TB and patient perspectives towards TB. Scale items were summed such that higher scores indicated higher levels of TB stigma. The Cronbach’s alpha value for the TB stigma scale was 0.90, indicating high internal consistency.

Alcohol stigma scale: Alcohol stigma was assessed with the Alcohol-Adapted Perceived Discrimination-Devaluation (PDD) scale [57] and five additional items developed by Kalichman and colleagues to assess the stigmatization of alcohol use among PWH [58]. The Alcohol-Adapted PDD is an instrument to assess an individual’s perceived alcohol stigma towards people who engage in unhealthy alcohol use. Seven PDD questions assessed perceived discrimination towards those who engage in unhealthy alcohol use and five questions assessed perceived devaluation. Perceived discrimination is when people feel they are purposefully being mistreated because of their alcohol use. Perceived devaluation is when an individual perceives themself to be less valued because of their alcohol use. The scale was summed such that higher scores denoted higher levels of perceived alcohol stigma. The Cronbach’s alpha value for the alcohol stigma scale was 0.79, indicating moderate internal consistency.

Intersectional stigma: We dichotomized each stigma scale using an approach described by Anvari and colleagues in South Africa [29] such that those who experienced stigmas higher than the median of the study population were considered to experience “high stigma”. In the rest of our descriptions, when we reference high stigma, we are referring to stigma levels above the median. To measure intersectional stigma, we created indicators of experiencing each pair of stigmas (HIV and TB only, HIV and alcohol only, TB and alcohol only) and an indicator of experiencing all three stigmas (0 otherwise).

Unhealthy alcohol use: Since we have previously found alcohol use to be under-reported in this population [50, 59], we used self-report combined with an alcohol biomarker, PEth, to categorize alcohol use as follows: “no/low alcohol use”: AUDIT-C < 3 and < 4 for women and men, respectively, and PEth < 50 ng/mL; “moderate alcohol use”: AUDIT-C ≥ 3 and ≥ 4 for women and men respectively and AUDIT-C < 6, or PEth ≥ 50 ng/mL and < 200 ng/mL; “unhealthy alcohol use”: AUDIT-C ≥ 6 or PEth ≥ 200 ng/mL. The highest category was used if self-report and PEth categories differed. The combination of PEth and AUDIT-C, both of which are highly specific, have been combined to increase sensitivity, given that neither self-report nor PEth are completely sensitive [60–62].

Covariates: Covariates of interest included participants’ age, gender, marital status, partner HIV status, household wealth as measured using a household asset index, and alcohol use. These variables were selected based on a priori knowledge and literature, as they have previously shown associations with TB, HIV, and unhealthy alcohol use [21, 29, 43] and with perceived health [63–66].

### Data Analysis

We conducted descriptive analyses of all variables overall and by gender. We calculated frequencies and proportions for categorical variables; we calculated medians with interquartile ranges (IQR) for continuous variables. Differences by gender were examined using Chi-squared or Kruskal-Wallis tests, as appropriate. Using unadjusted ordinal regression models, we separately considered the association between the following variables and poorer perceived general health status: indicators of experiencing high levels (scores above median) of each stigma (HIV, TB, and alcohol), indicators of experiencing each pair of stigmas (high levels of: (1) HIV and TB stigma, (2) TB and alcohol and stigma, (3) HIV and alcohol stigma), and experiencing high levels of all three stigmas (HIV, TB, and alcohol). In these analyses, we focused on intersectional stigma instead of on the effect of individual stigmas on poorer perceived health as these relationships are well established in the literature. We also fit multivariable ordinal regression models that controlled for age, gender, marital status, household assets, HIV status of partner, and alcohol use. We use the proportional odds model to estimate the average association between experiencing stigma and increased perceived health status. The odds ratio from this model reflects the average of the association between stigma and three binary outcome comparisons: (1) excellent vs. very good, good, or fair/poor, (2) excellent or very good vs. good or fair/poor, and (3) excellent, very good or good vs. fair/poor. To estimate the *average* association, we do not need to require the proportional odds assumption to be met. If the proportional odds assumption is met, we would further be able to conclude that the associations between stigma and each of the binary comparisons are equivalent; however, this is not the primary aim of our analysis. We also conducted multivariable models stratified by gender. Finally, we fit models that also included an interaction term between stigma and marital status to explore a possible effect of partner support on experiences of stigma and planned to explore stratified models if we found significant interaction (*p* < 0.10).

## Ethical Approvals

The study activities were approved by the Ethics review boards of Mbarara University of Science and Technology (MUST-2021-238), The Uganda National Council of Science and Technology (HS1962ES), and University of California San Francisco (21-34310). The participants gave written informed consent to participate in the study.

## Results

### Baseline Characteristics

Our sample included 379 participants who had data on the three stigma scales of interest (HIV, TB, and alcohol stigmas). In total, 121 TRAC participants were excluded: 116 did not have stigma scores because the stigma scales were not part of the original baseline survey but were added after the study began, and 5 were excluded due to missing/declined item responses. In a comparison of included versus excluded participants, there was a statistically significant difference by gender, but not by age or alcohol use. We believe this difference in gender has to do with the study recruitment technique. During recruitment, the research team initially recruited persons with lower-level alcohol use (based on previous study data) more quickly than persons with heavier alcohol consumption. We have previously observed that more of those with lower-level alcohol use are women, and the users who exhibited higher alcohol consumption are men in this setting [67]. The stigma questions were added later when recruitment was more focused on recruiting those with higher alcohol consumption.

The socio-demographic characteristics of participants are presented in Table 1. The median age of participants was 43 years (IQR: 37–50) and 60% were men. More than half of the sample was married (59%), among whom 65% reported that their spouse was living with HIV (i.e., they were in an HIV sero-concordant relationship). More men than women reported being married (72% vs. 40%, χ^2^ = 39.30, *p* < 0.001). About half (53%) of participants had high-risk alcohol use, with more men (69%) than women (28%) engaging in unhealthy alcohol use (χ^2^ = 76.75, *p* < 0.001). 12% (12%) described their health status as being fair or poor, with no significant differences between men and women.

**Table 1:** Participant characteristics for those with stigma scores, by gender, TRAC study (*n* = 379)

	Women	Men	Total	Test
	(*N* = 152)	(*N* = 227)	(*N* = 379)	
	N (%) or median (IQR)	N (%) or median (IQR)	N (%) or median (IQR)	Chi2 or Kruskal-Wallis (p-value)
Age	42.0 (36.0–50.0)	44.0 (37.0–50.0)	43.0 (37.0–50.0)	0.700 (0.403)
Married				39.297 (< 0.001)
Not married	92 (60.5%)	64 (28.2%)	156 (41.2%)	
Married	60 (39.5%)	163 (71.8%)	223 (58.8%)	
Spouse HIV status				46.1774 (< 0.001)
HIV-	11 (7.2%)	54 (23.8%)	65 (17.2%)	
HIV+	42 (27.6%)	103 (45.4%)	145 (38.3%)	
Unknown	7 (4.6%)	6 (2.6%)	13 (3.4%)	
Not married	92 (60.5%)	64 (28.2%)	156 (41.2%)	
Household wealth				2.6041 (0.272)
Low (bottom 40%)	58 (38.2%)	102 (44.9%)	160 (42.2%)	
Middle	59 (38.8%)	86 (37.9%)	145 (38.3%)	
High (top 20%)	35 (23.0%)	39 (17.2%)	74 (19.5%)	
Social Desirability Score	20.0 (17.0–22.0)	20.0 (17.0–22.0)	20.0 (17.0–22.0)	0.008 (0.930)
Alcohol use*				76.7454 (< 0.001)
No/low alcohol use	82 (53.9%)	33 (14.5%)	115 (30.3%)	
Moderate alcohol use	28 (18.4%)	37 (16.3%)	65 (17.2%)	
Unhealthy alcohol use	42 (27.6%)	157 (69.2%)	199 (52.5%)	
Perceived health status				2.0097 (0.570)
Excellent	47 (30.9%)	70 (30.8%)	117 (30.9%)	
Very good	33 (21.7%)	45 (19.8%)	78 (20.6%)	
Good	51 (33.6%)	89 (39.2%)	140 (36.9%)	
Fair/poor	21 (13.8%)	23 (10.1%)	44 (11.6%)	
TB stigma scale (possible range: 23–115)	111.0 (107.0–115.0)	111.0 (103.0–115.0)	111.0 (103.0–115.0)	1.239 (0.266)
HIV stigma scale (possible range: 10–50)	24.0 (18.0–32.5)	21.0 (14.0–29.0)	22.0 (17.0–30.0)	9.093 (0.003)
Alcohol stigma scale (possible range: 17–85)	67.0 (57.0–75.0)	61.0 (53.0–70.0)	63.0 (54.0–73.0)	8.609 (0.003)
High TB stigma (> 111 (median))				0.8664 (0.352)
No	79 (52.0%)	129 (56.8%)	208 (54.9%)	
Yes	73 (48.0%)	98 (43.2%)	171 (45.1%)	
High HIV stigma (> 22 (median))				2.4167 (0.120)
No	72 (47.4%)	126 (55.5%)	198 (52.2%)	
Yes	80 (52.6%)	101 (44.5%)	181 (47.8%)	
High alcohol stigma (> 63 (median))				5.5120 (0.019)
No	65 (42.8%)	125 (55.1%)	190 (50.1%)	
Yes	87 (57.2%)	102 (44.9%)	189 (49.9%)	
High TB and HIV stigma				3.9234 (0.048)
No	107 (70.4%)	180 (79.3%)	287 (75.7%)	
Yes	45 (29.6%)	47 (20.7%)	92 (24.3%)	
High TB and alcohol stigma				3.2631 (0.071)
No	109 (71.7%)	181 (79.7%)	290 (76.5%)	
Yes	43 (28.3%)	46 (20.3%)	89 (23.5%)	
High HIV and alcohol stigma				1.4039 (0.236)
No	109 (71.7%)	175 (77.1%)	284 (74.9%)	
Yes	43 (28.3%)	52 (22.9%)	95 (25.1%)	
High TB, HIV, and alcohol stigma				2.2400 (0.134)
No	128 (84.2%)	203 (89.4%)	331 (87.3%)	
Yes	24 (15.8%)	24 (10.6%)	48 (12.7%)	

*Alcohol use categories defined as follows: “no/low use” PEth < 50ng/ml and AUDIT-C negative; “moderate use” PEth 50-<200 or AUDIT-C positive and < 6; “unhealthy use” PEth ≥ 200 ng/ml or AUDIT-C ≥ 6. The highest category was used if self-report and PEth categories differed

**Fig. 1:**
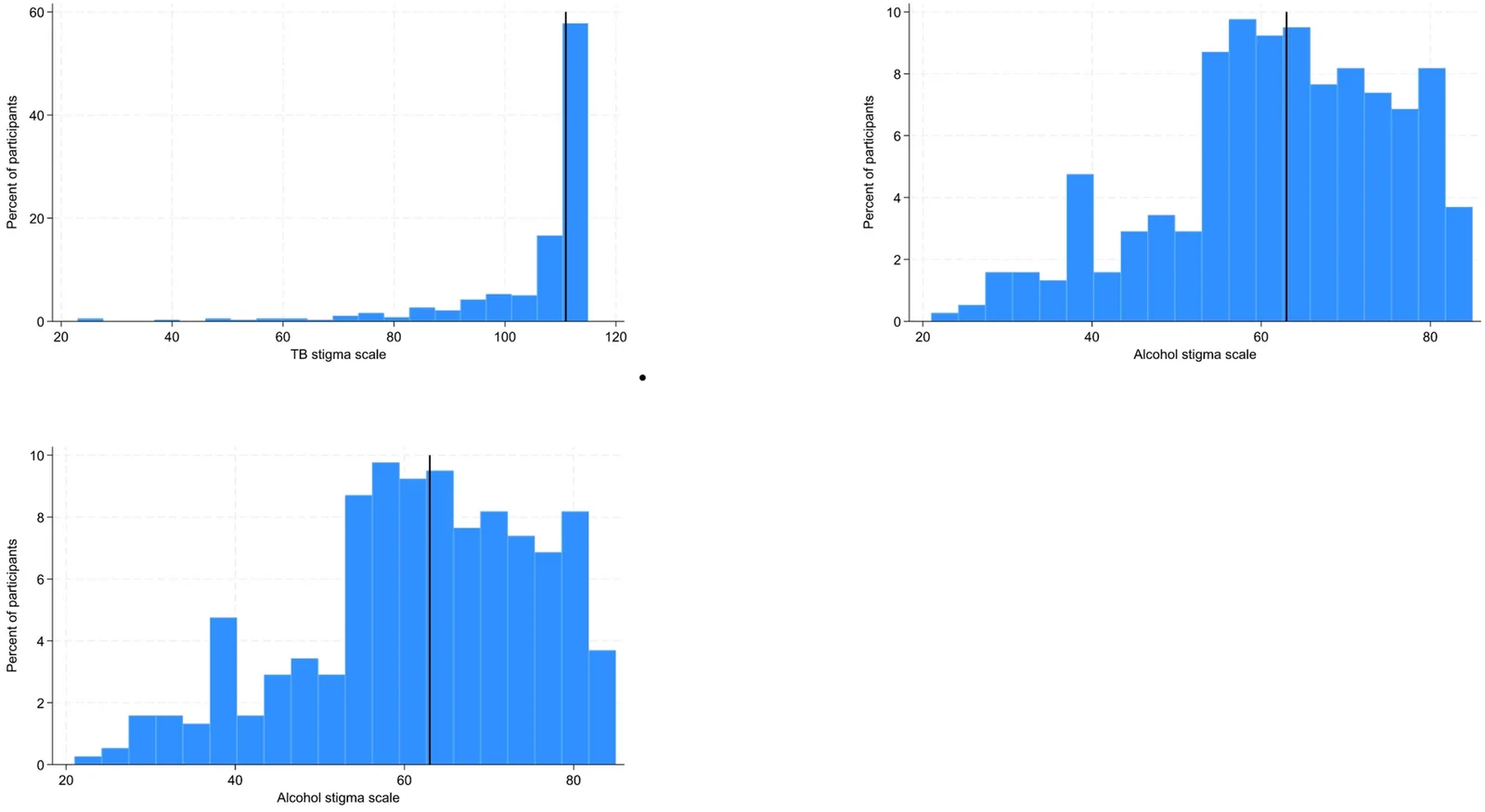
Histograms of TB stigma scale, HIV stigma scale, and alcohol stigma scale, with median lines

Stigma scores ranged widely (Fig. 1). The median HIV stigma score was 22 overall (IQR: 17–30, scale range of 10–50) with women reporting more HIV stigma than men (median 24 vs. 21, Kruskal-Wallis H = 9.09, *p* = 0.003) (Table 1). The median TB stigma score was 111 overall (IQR: 103–115, scale range of 23–115, no difference between men and women). The median alcohol stigma score was 63 overall (IQR: 54–73, scale range 17–85) with women reporting higher alcohol stigma scores than men (median 67 vs. 61, Kruskal-Wallis H = 8.61, *p* = 0.003). When we examined intersectional stigma, about a quarter of participants had high levels of two types of stigma (24% high HIV and TB stigma, 24% high TB and alcohol stigma, and 25% high HIV and alcohol stigma). There was also a statistically significant difference by gender in high TB and HIV stigma (30% women vs. 21% men, χ^2^ = 3.92, *p* = 0.048). 13% of participants reported high levels of all three (HIV, TB, and alcohol) stigmas.

### Unadjusted Models

In unadjusted models, we found no significant associations between any of the intersectional stigma combinations (e.g., high HIV and TB stigma, high HIV and alcohol stigma, high TB and alcohol stigma, or high HIV, TB, and alcohol stigmas) and poorer perceived health.

### Adjusted Models

In adjusted models, high intersectional HIV and alcohol stigma was associated with an increased odds of poorer perceived health (aOR = 1.62; 95% CI: 1.04–2.52; *p* = 0.03), but we found no significant associations between other HIV, TB, and alcohol stigma intersections with poorer perceived health (Table 2). We also fit adjusted models that included an interaction term between stigma and marital status (Suppl, Table 1). In these models, we found a significant interaction between intersectional HIV and alcohol stigma and marital status (Wald χ^2^ = 5.02, *p* = 0.03) such that those who were not married who also experienced both HIV and alcohol stigma had almost three times the odds of poorer perceived health (aOR = 2.79; 95% CI: 1.45–5.35; *p* < 0.01) compared to those who were not married and did not experience high HIV and alcohol stigma. We also fit an adjusted model that included an interaction term between intersectional HIV and alcohol stigma and gender but did not find a significant interaction (Wald χ^2^ = 1.25, *p* = 0.26). Finally, we ran an adjusted model that included a three-way interaction term between high intersectional HIV and alcohol stigma, marital status, and gender, but the interaction term was not statistically significant (Wald χ^2^ = 0.60, *p* = 0.44).

**Table 2:** Unadjusted and adjusted odds ratios (OR) and 95% confidence intervals (CI) for *poorer perceived* health, from separate ordinal logistic regression models (*n* = 379)

	OR (95% CI)	*p*-value	aOR (95% CI)	*p*-value
TB stigma scale (continuous, per 1 pt)	1.00 (0.99, 1.01)	0.70		
HIV stigma scale (continuous, per 1 pt)	1.06 (1.03, 1.08)	< 0.01		
Alcohol stigma scale (continuous, per 1 pt)	1.01 (1.00, 1.03)	0.06		
High TB and HIV stigma		0.38		0.22
No	1.00		1.00	
Yes	1.22 (0.79, 1.88)		1.33 (0.84, 2.10)	
High TB and alcohol stigma		0.99		0.88
No	1.00		1.00	
Yes	1.00 (0.65, 1.54)		1.04 (0.66, 1.62)	
High HIV and alcohol stigma		0.06		0.03
No	1.00		1.00	
Yes	1.52 (0.99, 2.33)		1.62 (1.04, 2.52)	
High TB, HIV, and alcohol stigma (all 3 scales)		0.39		0.27
No	1.00		1.00	
Yes	1.28 (0.73, 2.23)		1.39 (0.78, 2.47)	

Models adjusted forage, gender, marital status, household assets, HIV status of partner, and alcohol use

Due to the significant interaction between intersectional HIV and alcohol stigma and marital status, we then stratified models based on marital status. We found that high intersectional HIV and alcohol stigma was associated with increased odds of poorer perceived health among unmarried participants (aOR = 2.54; 95% CI: 1.33–4.86; *p* < 0.01) but not among married participants (aOR = 1.05; 95% CI: 0.56–1.95; *p* = 0.88; Table 3).

**Table 3:** Adjusted odds ratios (aOR) and 95% confidence intervals (CI) for lower perceived health status, from ordinal logistic regression models

Among unmarried participants only (n=156)								
	aOR (95% CI)	*p*-value	aOR (95% CI)	*p*-value	aOR (95% CI)	*p*-value	aOR (95% CI)	*p*-value
High TB and HIV stigma		0.58						
No	1.00		–		–		–	
Yes	1.21 (0.61, 2.39)		–		–		–	
High TB and alcohol stigma				1.00				
No	–		1.00		–		–	
Yes	–		1.00 (0.50, 2.01)		–		–	
High HIV and alcohol stigma						**< 0.01**		
No	–		–		1.00		–	
Yes	–		–		2.54 (1.33, 4.86)		–	
High TB, HIV, and alcohol stigma (all 3 scales)								0.12
No	–		–		–		1.00	
Yes	–		–		–		1.99 (0.83, 4.73)	

Among married participants only (n=223)								
	aOR (95% CI)	*p*-value	aOR (95% CI)	*p*-value	aOR (95% CI)	*p*-value	aOR (95% CI)	*p*-value
High TB and HIV stigma		0.22						
No	1.00		–		–		–	
Yes	1.47 (0.79, 2.75)		–		–		–	
High TB and alcohol stigma				0.80				
No	–		1.00		–		–	
Yes	–		1.08 (0.60, 1.95)		–		–	
High HIV and alcohol stigma						0.88		
No	–		–		1.00		–	
Yes	–		–		1.05 (0.56, 1.95)		–	
High TB, HIV, and alcohol stigma (all 3 scales)								0.98
No	–		–		–		1.00	
Yes	–		–		–		1.01 (0.47, 2.19)	

Models adjusted for age, gender, household assets, alcohol use, and HIV status of partner (married models only)

## Discussion

In this study of PWH on ART in southwestern Uganda, we found that intersectional HIV and alcohol stigma was associated with poorer perceived health. In addition, we found a significant interaction between HIV and alcohol stigma and marital status, suggesting that marriage or the social support that couples receive from their partners, may help to reduce feelings of poorer health even when people experience high intersectional HIV and alcohol stigma.

Our finding that intersectional HIV and alcohol stigma was associated with poorer perceived health adds to a growing body of literature that has found that intersectional HIV and alcohol stigma can have negative impacts on health, including mental health [29] and engagement with healthcare services [8, 10]. Stigma reduction interventions that use an intersectionality framework and aim to address multiple and co-occurring stigmas may help men and women to develop resiliency and build support in their lives to address stigma.

The significant interaction that we observed between HIV and alcohol stigma and marital status may mean that being married or having social support from a primary partner, can help reduce a sense of poor health even when stigma is present. Primary partners play a critical role in providing comfort and HIV-related social support and partners can help with engaging in behaviors to improve health [32, 34, 35, 68]. Interventions aimed at improving relationship dynamics such as trust, communication, closeness, and intimacy or partner support have been linked to positive health behaviors such as couple HIV counselling and testing [69, 70] and ART adherence [32, 71, 72]. In addition, the stress-buffering model suggests that partner-specific social support can protect against the negative impacts of stressors from outside the relationship, such as stigma [73]. Research also suggests that partner relationships can improve mutual emotional resilience [74]. In line with this, a recent study from Malawi found that supportive couple relationships buffer against the harms of anticipated HIV stigma on HIV treatment adherence [75]. Furthermore, a systematic review found that PWH with higher levels of social support were less likely to report HIV stigma, suggesting that receiving social support in general may also help reduce feelings of stigma [76]. It may be that social support, and not an intimate relationship, is the critical component that helps to reduce the effects of stigma. Furthermore, marital status is a rough proxy for social support. This finding should be considered exploratory and research that directly measures of social support, or relationship quality, are needed. Also, research that examines whether social support, from someone who is not an intimate partner, can help reduce intersectional stigma is needed. Interventions aimed at identifying a trusted contact who provides social support may also help to protect PWH from intersectional stigma.

We found no significant associations between any of the intersectional stigma combinations that included TB stigma (e.g., high HIV and TB stigma, high TB and alcohol stigma, or high HIV, TB, and alcohol stigmas) and poorer perceived health, even though the relative level of TB stigma was high. These results are not consistent with previous research, which has found associations and intersectional links between HIV and TB stigmas [19], between HIV diagnosis and TB stigma [20], and between unhealthy alcohol use and TB and HIV stigma [21].

This study had limitations. First, the data are baseline cross-sectional data so we cannot draw any conclusions about causality. To construct intersectional stigma variables, we dichotomized the stigma variables, which might have introduced significant bias into our analyses [77–79]. However, constructing a measure of intersectional stigma using continuous variables also presents challenges, the most important of which is that researchers must choose a cut off at which they believe stigma values are high enough to be considered intersectional. Instead, dichotomizing the stigma variables improves interpretability and allows for an easier definition of intersectional stigma. A strength of our analyses is that we did not dichotomize the outcome variable of poorer perceived health and instead used it as an ordinal variable. While there were some significant associations between HIV, TB, and alcohol stigmas independently with the outcome of poorer perceived health, our goal was to focus on the intersectional nature of these stigmas. Therefore, we did not focus on these stigmas individually in adjusted analyses. Also, based on the research questions of the parent study, the sample was comprised of PWH on ART, and participants were oversampled for alcohol use. Due to these eligibility criteria, the findings may not be representative of the general population of PWH, although alcohol use is common in Uganda. In addition, although the sample may not be representative, the health of those who report heavy alcohol use is of concern, and this sample allows a closer look at this population of interest. Therefore, oversampling for alcohol use may have increased our ability to detect associations between alcohol stigma and perceived health. In these analyses, we opted not to examine depression as an outcome as it is a mental health symptom and is usually correlated with other forms of stigma. In our analyses, we included a small number of possible confounders, choosing to focus on the relationship between intersectional stigma and perceived health status. There may be residual confounding or other variables that could be mediators of the relationship between intersectional stigma and perceived health. Future research could examine depression, viral load, or time on ART as potential mediators of this relationship. Another strength of our approach was that instead of only using self-reports of alcohol use, we used measurement of PEth combined with self-report to minimize issues of under reporting and achieve a more accurate measure of alcohol use in this population [60, 80, 81].

## Conclusion

Intersectional HIV and alcohol stigma was associated with poorer perceived health among PWH on ART. Interventions that address the intersectional nature of HIV and alcohol stigma in this population may help both women and men better cope with stigma and its negative consequences. Given our finding that marital status may buffer the effects of HIV and alcohol stigma on poorer perceived health, research that seeks to understand the mechanisms by which intimate relationships help protect partners from the effects of stigma on health may help to identify specific areas that can be targeted in interventions for unmarried individuals in order to help them develop social support and resilience. Future qualitative research exploring the mechanisms by which intimate relationships mitigate the impacts of intersectional stigma on health can help inform these interventions.
